# Mutation analysis, treatment and prenatal diagnosis of Chinese cases of methylmalonic acidemia

**DOI:** 10.1038/s41598-020-69565-z

**Published:** 2020-07-27

**Authors:** Chuan Zhang, Xing Wang, Shengju Hao, Qinghua Zhang, Lei Zheng, Bingbo Zhou, Furong Liu, Xuan Feng, Xue Chen, Panpan Ma, Cuixia Chen, Zongfu Cao, Xu Ma

**Affiliations:** 1 Graduate School of Peking Union Medical College, Beijing, 100730 China; 2 Gansu Province Medical Genetics Center, Gansu Province Maternal and Child Health Care Hospital, Lanzhou, 730050 China; 3 National Research Institute for Family Planning, National Human Genetic Resources Center, No. 12 Dahuisi Road, Beijing, 100081 China

**Keywords:** Diseases, Medical research, Molecular medicine

## Abstract

Methylmalonic acidemia (MMA)-affected patients may have developmental, hematological, neurological, metabolic, ophthalmological, and dermatological clinically abnormal findings. This study aimed to identify mutations in 13 Chinese MMA cases. We provided genetic counseling, treatment, and prenatal diagnosis for the families with MMA. Liquid chromatography-tandem mass spectrometry (LC–MS/MS) was performed and the results were confirmed by gas chromatography and mass spectrometry (GC/MS). Variant screening in probands was performed by targeted next-generation sequencing. Identified variants were confirmed by Sanger sequencing. Of these 13 MMA cases, seven were isolated MMA, and among them, six were caused by variants in *MMUT* and one was caused by a variant in *MCEE*. The other six cases were MMA with homocystinuria, which was caused by variants in *MMACHC*. We found six novel variants in three MMA-causing genes as follows: c.2008G>A, c.301_302insTA, c.984delC, and c.319A>T of *MMUT*; c.445T>C of *MMACHC*; and c.296T>C of *MCEE*. We provided prenatal diagnosis for two families with MMA at their next pregnancy, and one family had a healthy newborn. In conclusion, our findings expand the spectrum of genotypes in MMA. Effective genetic counseling is required to allow awareness of the patients’ families that MMA disease is treatable and a good prognosis can be obtained.

## Introduction

Methylmalonic acidemia is a group of inborn errors of metabolism causing multisystem disease. MMA has two subtypes of isolated MMA and MMA with homocystinuria. Two genetic defects of deficiency of methylmalonyl CoA mutase and defects in the synthesis of the coenzyme adenosylcobalamin cause isolated MMA^1^. The worldwide estimated incidence of MMA is approximately 1/48,000–1/250,000^2^. The incidence of MMA is different among the different MMA subtypes and regions of China. The total incidence of MMA in China is approximately 1: 28,000 for MMA at birth^3^, but in Shandong Province, the incidence of cobalamin (cbl) C type is approximately 1: 3,920 live births^4^. According to the age of onset, MMA can be divided into two forms as follows. In the early-onset form, patients with MMA present with symptoms within the first year. The late-onset form is rarer than the early-onset form and is easily misdiagnosed or missed. In the late-onset form, patients present with a milder clinical phenotype with acute or slowly progressive nervous system symptoms and behavioral disorders at any time from childhood to adulthood^5^. The clinical manifestations of patients with MMA are complex and varied, range in severity, and can manifest as an acute or chronic course. Severe cases of MMA and death occur in the neonatal period, and mild disease can occur in adulthood.

MMA is a genetically heterogeneous disease. Isolated MMA can be caused by mutations in *MMUT* (OMIM, 609058), which result in deficiency of methylmalonyl CoA mutase, and this form is unresponsive to vitamin B12 therapy. Mutations in *MCEE* lead to deficiency of methylmalonyl-CoA epimerase, which can result in mild isolated MMA^6^. Other forms of isolated MMA are found in a subset of patients with defects in synthesizing the coenzyme adenosylcobalamin and are classified into three groups: cblA (OMIM, 251100), which is caused by mutations in *MMAA* (OMIM, 607481); cblB (OMIM, 251110), which is caused by mutations in *MMAB* (OMIM, 607568)^7^; and cb1D variant 2, which is caused by mutations in *MMADHC* (OMIM, 611935). The finding of biallelic pathogenic variants in one of the five genes (*MMUT*, *MMAA*, *MMAB*, *MCEE*, and *MMADHC*) associated with isolated MMA with confirmation of carrier status in the parents can establish the diagnosis of MMA. Approximately 97% of isolated MMA cases were caused by mutations in *MMUT*, *MMAA*, and *MMAB*, and mutations in *MCEE* and *MMADHC* only account for approximately 1–3%^6,8^. MMA with homocystinuria can be found in the following complementation groups: cblC (OMIM, 277400), which is caused by mutations in *MMACHC* (OMIM, 609831); cblD (OMIM, 277410), which is caused by mutations in *MMADHC* (OMIM, 611935); and cblF (OMIM, 277380), which is caused by mutations in *LMBRD1* (OMIM, 612625)^9^.

In our study, we recruited 13 unrelated patients with MMA and studied the clinical, biochemical, and hereditary pathogenesis of these patients. We performed accurate genetic metabolic disease screening, molecular diagnosis, and genetic counseling for these families. We provided invasive prenatal diagnosis for two families and followed up their pregnancy results.

## Patients and methods

### Patients

A total of 13 unrelated patients with MMA (seven with isolated MMA and six with MMA with homocystinuria) and their families were recruited. The patients were from Gansu Provincial Maternal and Child Health Care Hospital during November 2016 to May 2019. The age of the probands ranged from 1 day to 5 years old. All patients were from non-consanguineous families. This study was performed according to the tenets of the Declaration of Helsinki. This study was approved by the Ethics Committee of Gansu Provincial Maternal and Child Health Care Hospital (No. 4 of the hospital ethics review, 2016). Written informed consent was obtained from all participants in this study, and written informed consent of patients younger than 18 years old was obtained from their parents.

## Methods

### Genetic metabolic disease screening

We collected dried blood spots (in whole blood) of the probands. Blood amino acids, free carnitine, and acylcarnitines were measured by using liquid chromatography-tandem mass spectrometry (LC–MS/MS) (TQD; Waters, USA). The reference levels of propionylcarnitine (C3) and C3/acetylcarnitine (C2) in the blood were 0.3–4.95 μmol/L and 0.05–0.27 μmol/L, respectively. Concentrations of organic acids in urine were measured by gas chromatography and mass spectrometry (GC/MS) (GCMS-QP 2010; Shimadzu Corporation, Japan) in these patients. The normal value of urine methylmalonic acid ranges from 0.0–5.34 μmol/L.

### Treatment and follow up

Metabolic therapy, including a high-calorie diet with special formula supplementation (no isoleucine, threonine, methionine, and valine), stopping protein intake and intramuscular cobalamin were provided to most patients. Treatment for the patients was then adjusted according to their response to vitamin B12 and other individual conditions^7,10–12^.

Two families had an invasive prenatal diagnosis during 19–20 weeks of pregnancy. We obtained 15 ml of amniotic fluid and then used Sanger sequencing to detect the same mutations as those found in the probands.

### Genomic DNA preparation

A total of 2–3 ml of blood samples were collected from the probands and their parents. Genomic DNA was extracted using the Tiangen Biotech DNA extraction kit (Beijing, China).

### Targeted next generation sequencing and Sanger Sequencing

Mutation screening for the probands was performed by targeted gene capturing and sequencing of 175 related genes by MyGenostics Corporation (MyGenostics GenCap Enrichment Technologies, Beijing, China).The list of genes is shown in Table S1. Candidate variants were confirmed in the parents in each family by Sanger sequencing. Primers for polymerase chain reaction were designed by Primer3 Input (v.0.4.0, https://bioinfo.ut.ee/primer3-0.4.0/) (Table S2). Amplification conditions for Sanger sequencing are shown in Table S2. DNA sequencing was performed using an ABI 3500DX Genetic Analyzer (Applied Biosystems, USA).

### Bioinformatics analysis

The variants were described according to the nomenclature recommended by the Human Genomic Variation Society. Novel variants were checked in the Human Gene Variant Database (HGMD) (https://www.hgmd.cf.ac.uk/) and ClinVar database (https://www.ncbi.nlm.nih.gov/clinvar/). We used PolyPhen2 (https://genetics.bwh.harvard.edu/pph2) and PROVEAN (https://provean.jcvi.org/index.php) tools to predict the possible functional role of novel variants. InterVar software (https://wintervar.wglab.org/) was used to evaluate the pathogenicity of the novel variants with reference to the standards and guidelines of the American College of Medical Genetics and Genomics (ACMG)^13^. This process has been described previously^14,15^.

### Ethics approval and consent to participate

This study was approved by the Ethics Committee of the Gansu Provincial Maternal and Child Health Care Hospital (No. 4 of hospital ethics review, 2016). Written informed consents have been obtained from all participants the study procedure started.

## Result

### Clinical features, biochemical indicators and variant analysis

Our 13 MMA cases from 13 unrelated families were recruited from the genetic counseling clinic and the neonatal intensive care unit of Gansu Provincial Maternal and Child Health Care Hospital. Two of the patients had the late-onset form (> 1 year old) and 11 had the early-onset form (< 1 year old). The clinical features and biochemical indicators are shown in Table 1. Seven of the cases were isolated MMA and six were MMA with homocystinuria.

**Table 1:** Novel mutations of *MUT*, *MMACHC* and *MCEE.*

Gene	Nucleotide change	Amino acid Change	PolyPhen2 result (Score)	PROVEN result (Score)	Pathogenicity	Evidence
*MUT*	c.2008G>A	p.G670S	PD (1.000)	D (− 5.850)	LP	PM1 PM2 PM5 PP3
	c.301_302insTA	p.T101Ifs*80	NA	NA	P	PVS1, PS2 and PM2
	c.984delC	p.W329Gfs*4	NA	NA	P	PVS1, PS2 and PM2
	c.319A>T	p.I107F	PD (1.000)	D (− 3.950)	LP	PM1 PM2 PP3 PP4
*MMACHC*	c.445T>C	p.C149R	PD (0.884)	D (− 7.990)	P	PM1 PM2 PP3 PP4
*MCEE*	c.296T>C	p.L99P	PD (1.000)	D (− 6.950)	LP	PM1 PM2 PP3 PP4

*PD* Probably Damaging, *D* Deleterious, *P* Pathogenic, *LP* Likely pathogenic.PolyPhen2 result: The score is closer to 1, the damaging will be more strong; Proven Result: Variants with a score equal to or below − 2.5 are considered “deleterious”, Variants with a score above − 2.5 are considered “neutral”. The type of evidence refers to. ACMG/AMP 2015 guideline (https://wintervar.wglab.org/).

**Figure 1:**
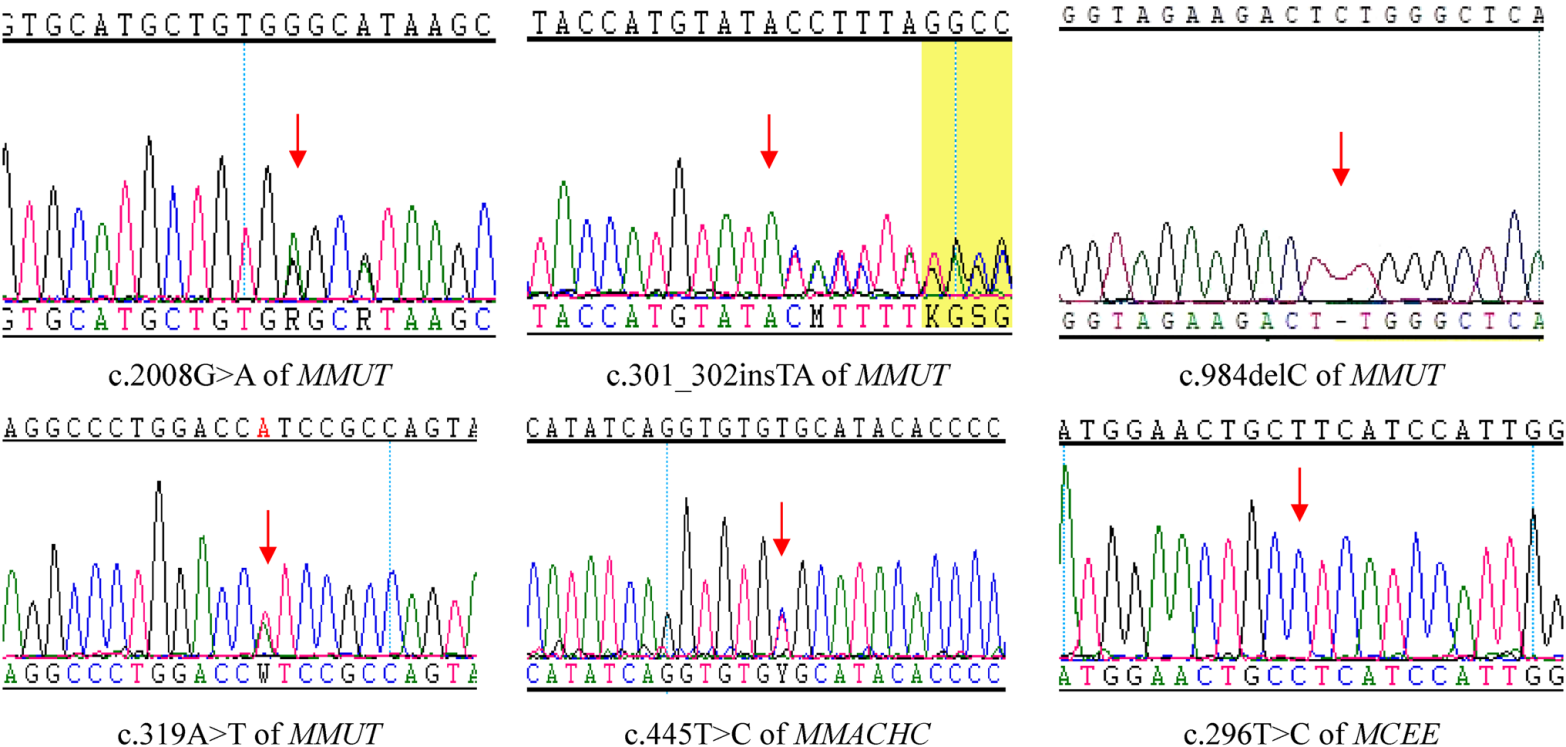
Novel variants of *MMUT*, *MMACHC*, *MCEE*.

Variants in *MMUT*: MMA was caused by a homozygous variant in one patient and MMA was caused by compound heterozygous variants in *MMUT* in five patients. Eleven different variants of *MMUT* were identified in these six patients (Table 2). Eight were missense variants and three were frame-shift variants. Seven of the variants were previously reported^11,16–20^ and four variants, c.2008G>A (p.G670S), c.301_302insTA(p.T101Ifs*80), c.984delC (p.W329Gfs*4), and c.319A>T(p.I107F), were not been previously reported in the HGMD and ClinVar databases (Table 2, Fig. 1).

**Table 2:** Results of DQ/IQ tests of the 5 patients.

Case No	Age at test	DQ					Full-scale IQ
		Gross motor	Fine motor	Language	Adaptability	Personal-social	
2	2Ys/5Ys	75	69	68	63	64	62
7	8Mons	72	63	78	62	59	
8	5Ys/7Ys	/	/	/	/	/	54/48
9	3Ys	78	75	72	77	69	
13	10Mons/2Ys	68/76	95/61	95/61	95/60	95/61	

*Mon* month, *D* day, *Y* Year.

**Figure 2:**
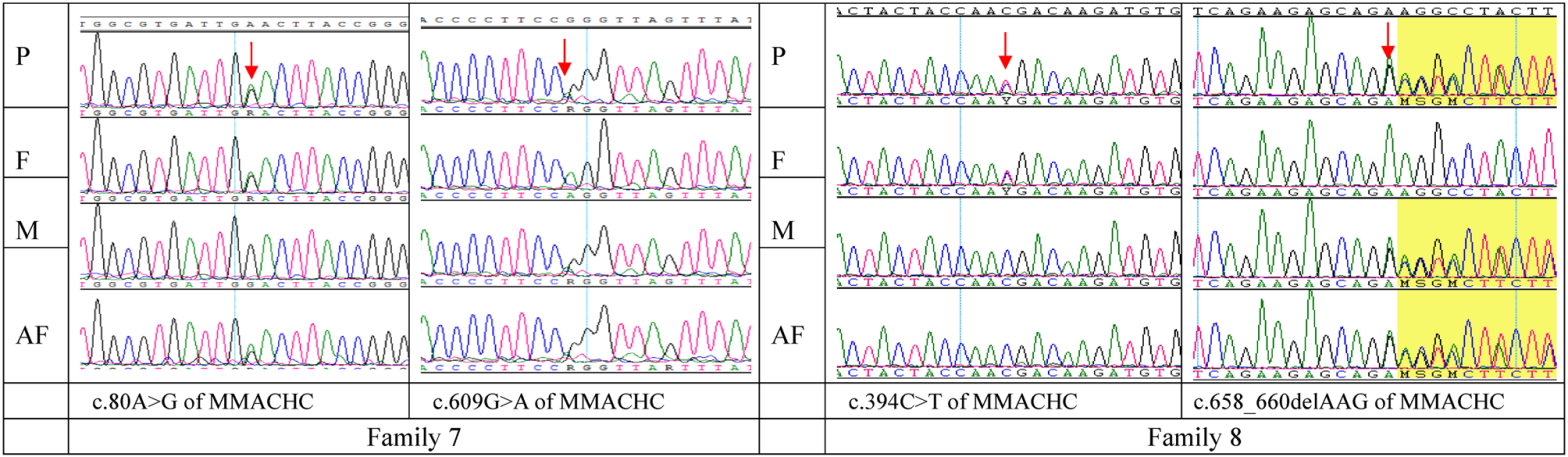
Prenatal diagnosis of MMA families. *P* proband, *F* father, *M* mother, *AF* amniotic fluid.

Variants in *MMACHC*: MMA was caused by a homozygous variant in two patients and MMA was caused by a compound heterozygous variant in *MMACHC* in four patients. Six types of variants in *MMACHC* were detected in these six patients (Table 2). Two missense variants, two frame-shift variants, and two nonsense variants were identified. Among them, c.445T>C (p.C149R) was not been previously reported in the HGMD and ClinVar databases (Table 2, Fig. 1). Two families with MMA caused by variants in *MMACHC* chose prenatal diagnosis through amniotic fluid puncture at their next pregnancy. The fetus from family 7 had the same compound heterozygous variant detected as that in the proband, and the fetus from family 8 only had a maternal heterozygous variant and there was no paternal variant(Fig. 2).

Variants in *MCEE*: One patient with MMA had the homozygous variant c.296T>C(p.L99P) in *MCEE* (Table 2, Fig. 1). This variant has not been previously reported.

Bioinformatics analysis: We used PolyPhen2 and PROVEAN tools to predict the possible functional role of these six novel variants. According to the ACMG guidelines and InterVar software, these six novel variants were categorized as “likely pathogenic” (Table 2).

### Follow up

Because of severe metabolic acidosis, eight families (numbers 1, 3, 4, 5, 6, 10, 11, and 12) abandoned treatment for the probands, and these patients eventually died. Family 7 provided treatment for the patient. However, the patient had a poor vitamin B12 response, and the patient died of metabolic acidosis at 11 months because of poor treatment. Patients 2 and 13 received oral L-carnitine, vitamin B12, and folate. Patients 8 and 9 received oral L-carnitine, betaine, vitamin B12, and folate (Table 3). One month later, the levels of serum C3 and C3/C2, urine MMA, and serum homocysteine (HCY) were slightly decreased. Six months later, serum C3, C3/C2, and HCY levels, and urine MMA levels of the patients were in the normal range (Table 3).

**Table 3:** Clinical characteristics, diagnosis, genotype information, treatment and prognosis of MMA patients in this study.

Case no	Age of onset	Curr-ent age	Sex	Blood C3 0–1–6 month (normal: 0.3–4.95 μmol/L)	Blood C3/C2 0–1–6 month (normal: 0.05–0.27)	Urinary MMA 0–1–6 month (normal: 0.0–5.34 μmol/L)	HCY 0–1–6 month (normal: 0.0–15.0 μmol/L)	Treatment (L-carnitine, betaine, B12 and folate)	Methylcobal-amin (B12) response	Prognosis	Gene	Genotype
1	14 Ds	/	F	10.2	0.79	391.1	7.5	No	/	Dead	*MUT*	c.2008G>A /c.103C>T
2	2 Ys	5Y	F	12.11/2.98/2.19	0.7/0.23/0.09	242.15/31.87/4.98	6.3	Yes	Effective	DD	*MUT*	c.301_302insTA /c.613G>A
3	6 Ds	/	M	10.59	0.78	403.59	4.3	No	/	Dead	*MUT*	c.984delC/ c.984delC
4	1 D	/	M	11.28	1.38	512.3	3.5	No	/	Dead	*MUT*	c.1106G>A /c.323G>A
5	2 Ds	/	M	11.35	1.63	1,117.47	5.3	No	/	Dead	*MUT*	c.914 T>C/ c.494A>G
6	1 D	/	M	13.2	0.45	300.46	2.2	No	/	Dead	*MUT*	c.1038_1040delTCT/ c.319A>T
7	8 Mons	/	F	14.83	0.69	334.5	63.9	No	Ineffective	Dead	*MMACHC*	c.80A>G /c.609G>A
8	5 Ys	7Y	F	8.87/3.35/1.27	1.77/0.19/0.14	149.86/36.09/3.25	65.4/23.2/8.9	Yes	Effective	DD	*MMACHC*	c.394C>T /c.658_660delAAG
9	56 Ds	3Y	M	8.98/3.48/2.15	0.84/0.21/0.18	358.2/6.02/2.98	62.1/52.5/6.8	Yes	Effective	DD	*MMACHC*	c.609G>A/ c.609G>A
10	15 Ds	/	M	10.32	1.01	431.2	47.8	No	/	Dead	*MMACHC*	c.609G>A/ c.609G>A
11	3 Mons	/	M	11.64	0.62	175.85	41.1	No	/	Dead	*MMACHC*	c.445 T>C/ c.609G>A
12	40 Ds	/	M	9.94	0.89	331.2	54.3	No	/	Dead	*MMACHC*	c.80A>G /c.567dupT
13	7 Mons	3Y	F	14.38/6.42/3.27	0.8/0.31/0.16	522/81.74/3.63	4.9	Yes	Effective	DD	*MCEE*	c.296 T>C/ c.296 T>C

Mon: month; D: day; Y: Year; M: male; F: female; DD: development delay.

The developmental quotient (DQ) was evaluated using the Revised Gesell Developmental Evaluation for children (< 4 years). This evaluation provides a developmental profile in five domains, including adaptive, gross motor, fine motor, language, and personal-social domains^9^. If the DQ of a child in any specific domain was ≤ 75^9^, development was below average. The intelligence quotient (IQ) was assessed by the Wechsler Intelligence Scale for Children (> 4 years). Low intelligence was defined as an IQ score < 80^9^. We evaluated the DQ for four patients (patient numbers 2, 7, 9, 13) and the IQ for patient 8 (Table 2). The patients from families 2 and 8 were treated too late, which resulted in mental retardation and motor dysfunction. Patient 9 had a good vitamin B12 response. However, because of a lack of timely treatment, he still had a certain degree of neurodevelopmental delay. He is currently 3 years old and his language is also slightly delayed. Patient 13 achieved better treatment results, but unfortunately, because of the COVID-19 pandemic, continuous treatment was interrupted, and she is currently in a serious condition.

Families 7 and 8 had prenatal diagnosis during pregnancy. The same compound heterozygous variants as those in the proband were found in the fetus of family 7, and this family stopped the pregnancy (Fig. 2). The fetus of family 8 only carried the maternal heterozygous variant (Fig. 2), and this family chose to continue the pregnancy with successful delivery.

## Discussion

In this study, we investigated the genetic etiology for 13 unrelated patients with MMA by using molecular testing. Six patients had MMA caused by homozygous/compound heterozygous variants in *MMUT*, 11 types of variants were found, and four of these variants were novel variants. Six patients had MMA caused by variants in *MMACHC*, six different variants were found, and c.445T>C was a novel variant. One patient had MMA caused by a novel homozygous variant, c.296T>C, in *MCEE*.

In our study, we found the six novel variants c.2008G>A, c.301_302insTA, c.984delC, and c.319A>T in *MMUT*, c.445T>C in *MMACHC,* and c.296T>C in *MCEE*. By using PolyPhen2 and PROVEAN tools and InterVar software, and according to the ACMG/AMP guidelines, these six novel variants were categorized as “likely pathogenic” (Table 2). With regard to the c.2008G>A(p.G670S) variant of *MMUT*, the same amino acid position variant p.G670R was reported in a 7-day-old female neonate^21^, but p.G670S has not been reported previously.

In our study, all of the six patients with MMA with homocystinuria had variants in *MMACHC*. The frequency of the variants in *MMACHC* was associated with the genetic background of the patients. In our patients, c.609G>A was the most frequent variant (50%, 6/12), which is consistent with reports in the northern Chinese population^20^. However, the frequency of this variant is not high in the European population^22^. Lerner-Ellis et al.^22^ reported that the variant c.271dupA accounted for 40% of all disease alleles in the European population, but we did not find this variant in our patients. Liu et al.^20^ showed that the frequency of c.271dupA was low (1/140). The c.609G>A, c.658_660delAAG, c.482G>A, c.394C>T, and c.80A>G mutations are the most common mutations in Chinese people, and in European people, the most common mutations are c.271dupA, c.331C>T, and c.394C>T. The variant c.394C>T is common in both European and Chinese people. Lerner-Ellis et al^22^ reported that the variant c.394C>T was associated with late-onset MMA. However, in Liu et al.’s study^20^, eight patients carried the variant c.394C>T, and only two patients had late-onset MMA. Therefore, we consider that the variant c.394C>T is also associated with early-onset MMA.

In the seven patients with isolated MMA, six were caused by variants in *MMUT* and one was caused by a homozygous variant in *MCEE*. Only approximately 21 patients with isolated MMA have been reported to have MMA caused by variants in *MCEE*^23–29^. To the best of our knowledge, we report the first patient with MMA caused by a variant in *MCEE* in Chinese people, and this was caused by the novel homozygous variant c.296T>C. We did not perform functional analysis of this novel variant at the cellular and animal levels. However, we used function prediction software to access the pathogenicity of this variant, and it was categorized as a “likely pathogenic” variant.

The girl with *MCEE* revisited the hospital at 7 months old because of a positive LC–MS/MS result, and she had genetic diagnosis and treatment at 10 months old. During the re-examination, LC–MS/MS and GCMS indicated MMA. A comprehensive evaluation of our patient was normal. The gross motor DQ was 68, the fine motor DQ was 95, the language DQ was 95, the adaptability DQ was 95, and the personal-social DQ was 95. Oral L-carnitine and folate, and intramuscular injection of methylcobalamin led to a reduction in C3 and C3/C2 levels. Unfortunately, because of the COVID-19 pandemic, the patient’s parents did not insist on continuous treatment, and currently, the patient is in a serious condition. Currently, the patient is 2 years old. A comprehensive evaluation of this patient showed retardation, the gross motor DQ was 76, the fine motor DQ was 61, the language DQ was 61, the adaptability DQ was 60, and the personal-social DQ was 61. Abily-Donval et al.^28^ summarized the treatment prognosis of eight patients with *MCEE*^23–27^. After suitable treatments, growth and psychomotor development of four patients were satisfactory, and their development was normal. The other four patients also had suitable treatment, but they still had problems in psychomotor, language, and nervous system development. Because our patient discontinued treatment, unfortunately, we do not have sufficient experience for treating patients with *MCEE*.

All of the patients with MMA were from the same Province, Gansu, which is located in the northwest of China. This region is economically insular with limited population mobility. MMA was caused by a homozygous variant in *MMUT* in one patient, by homozygous variants in *MMACHC* in two patients, and by a homozygous variant in *MCEE* in one patient. Although these patients were born from non-consanguineous families, we suspect that these homozygous cases might be due to a founder mutation in the local population.

In recent years, an increasing number of provinces in China have carried out newborn screening for genetic metabolic disorders by using MS/MS and GC/MS. With the development of molecular diagnostic technology and a decrease in cost of tests, currently, next-generation sequencing is a more effective method of providing accurate molecular diagnosis for some rare diseases^30^. Therefore, we can provide accurate and rapid diagnosis for patients who are affected by genetic metabolic disorders. We can also provide genetic counseling for the families and prenatal diagnosis for their next child. In our study, we provided prenatal diagnosis for two families with MMA caused by variants in *MMACHC*. The fetus from family 7 carried the same variants as those in the proband, and this family terminated the pregnancy. Intrauterine therapy is used to treat fetuses with MMA. Trefz et al.^31^ reported a patient with MMA in whom prenatal maternal treatment from week 15 of pregnancy prevented disease manifestation in a girl who is currently 11 years old with a normal IQ. However, we did not offer prenatal B12 therapy to the mother of family 7, and the pregnant woman chose to terminate the pregnancy. The fetus from family 8 only carried the maternal heterozygous variant, and this child was successfully delivered. Newborn screening at 72 h and biochemical examinations after 1 month were normal.

In our 13 patients with MMA, nine (69.2%) of them were detected by newborn screening through MS/MS and GC/MS. Four (30.8%) of these patients were diagnosed on the basis of clinical symptoms and biochemical examinations, including elevated serum C3 and C3/C2 levels and urine MMA levels, and MMA was confirmed by molecular testing. Unfortunately, only five patients received treatment. While four patients are currently still alive, the chance of recovery of these patients is not good.

## Conclusion

We studied 13 patients with MMA from the northwest of China, and by using a molecular test, we identified their genetic etiology. We found six novel variants in three MMA-causing genes, *MMUT*, *MMACHC,* and *MCEE*. We provided genetic counseling for these families with MMA, and two families had prenatal diagnosis during their next pregnancy. Our findings extend the mutation spectrum of isolated MMA in the Chinese population. Newborn screening and next-generation sequencing can provide an accurate diagnosis for genetic metabolic diseases at an early age. However, more effective genetic counseling, and timely treatment and follow-up are required to make the patients’ families aware that some genetic metabolic diseases are treatable and can achieve a good prognosis.

## Supplementary information

Supplementary file1 (DOC 71 kb)

## Data Availability

All data analysed during this study are included in this published article.
