# Supplementary material for: Mutation analysis, treatment and prenatal diagnosis of Chinese cases of methylmalonic acidemia
Source: Sci Rep. 2020 Jul 27;10:12509. doi: 10.1038/s41598-020-69565-z (PMC7385101; doi:10.1038/s41598-020-69565-z)
Supplement: Supplementary file 1 — Supplementary file1 (DOC 71 kb) [file 41598_2020_69565_MOESM1_ESM.doc]

**Mutation analysis, treatment and prenatal diagnosis of Chinese cases of methylmalonic acidemia**

Chuan Zhang^1,2,3^, Xing Wang ^2^, Shengju Hao^2^, Qinghua Zhang^2^, Lei Zheng^2^, Bingbo Zhou^2^, Furong Liu^2^, Feng Xuan^2^, Xue Chen^2^, Panpan Ma^2^, Cuixia Chen^1,3^, Zongfu Cao^1,3*^, Xu Ma^1,3*^

*1Graduate School of Peking Union Medical College, Beijing, 100730, China.;2Gansu Province Medical Genetics Center, Gansu Province Maternal and Child Health Care Hospital, Lanzhou, 730050, China;3National Research Institute for Family Planning, National Human Genetic Resources Center, Beijing, 100081, China.*

*Corresponding Author:Dr. Zongfu Cao(zongfu_cao@163.com) and Xu Ma (maxubioinfo@163.com),No. 12 Dahuisi Road, Beijing, 100081.

Table S1. Genes in the genetic metabolic diseases panel.

| *AASS* | *ASL* | *DBT* | *GATM* | *HSD17B3* | *MOCS2* | *PNPO* | *SLC6A19* |
| --- | --- | --- | --- | --- | --- | --- | --- |
| *ABAT* | *ASPA* | *DDC* | *GCDH* | *HSD3B2* | *MPV17* | *POLG* | *SLC6A20* |
| *ABCD4* | *ASS1* | *DGUOK* | *GCH1* | *IDH2* | *MTHFR* | *PRODH* | *SLC6A8* |
| *ABHD5* | *ATP7A* | *DHFR* | *GCSH* | *INPP5E* | *MTR* | *PSAT1* | *SLC7A7* |
| *ACAD8* | *ATP7B* | *DHTKD1* | *GLDC* | *IVD* | *MTRR* | *PTPN11* | *SLC7A9* |
| *ACADM* | *AUH* | *DLD* | *GLUD1* | *KDM6A* | *MUT* | *PTS* | *SOX9* |
| *ACADS* | *BCAT1* | *ERCC6* | *GLUL* | *KMT2D* | *MVK* | *QDPR* | *SPR* |
| *ACADSB* | *BCAT2* | *ERCC8* | *GLYCTK* | *L2HGDH* | *NAGLU* | *RRM2B* | *SRD5A2* |
| *ACADVL* | *BCKDHA* | *ETFA* | *GNMT* | *LAMP2* | *NAGS* | *SARDH* | *SRY* |
| *ACAT1* | *BCKDHB* | *ETFB* | *GNS* | *LMBRD1* | *NR0B1* | *SERAC1* | *StAR* |
| *ACSF3* | *BTD* | *ETFDH* | *GPHN* | *MAOA* | *NR5A1* | *SGSH* | *SUCLA2* |
| *ADK* | *CBS* | *ETHE1* | *HADH* | *MAT1A* | *OAT* | *SLC19A1* | *SUCLG1* |
| *AHCY* | *CDKL5* | *FAH* | *HADHA* | *MCCC1* | *OGDH* | *SLC22A5* | *SUOX* |
| *ALDH4A1* | *CPS1* | *FBXL4* | *HADHB* | *MCCC2* | *OPA3* | *SLC25A13* | *TAT* |
| *ALDH5A1* | *CPT1A* | *FH* | *HAL* | *MCEE* | *OTC* | *SLC25A15* | *TAZ* |
| *ALDH6A1* | *CPT2* | *FOLR1* | *HCFC1* | *MECP2* | *PAH* | *SLC25A20* | *TH* |
| *ALDH7A1* | *CTH* | *FOLR2* | *HGD* | *MLYCD* | *PC* | *SLC25A4* | *TK2* |
| *ALPL* | *CYP11B1* | *FOXG1* | *HGSNAT* | *MMAA* | *PCBD1* | *SLC2A1* | *TWNK* |
| *AMT* | *CYP17A1* | *FTCD* | *HLCS* | *MMAB* | *PCCA* | *SLC2A2* | *TYMP* |
| *AR* | *CYP21A2* | *G6PD* | *HMGCL* | *MMACHC* | *PCCB* | *SLC36A2* | *UROC1* |
| *ARG1* | *D2HGDH* | *GALK1* | *HPD* | *MMADHC* | *PHGDH* | *SLC3A1* | *WT1* |
| *ARX* | *DBH* | *GAMT* | *HSD17B10* | *MOCS1* | *PNPLA2* | *SLC46A1* |  |

Table S2 Primers and PCR conditions for Sanger sequencing

| Gene | Location | Sequence(5’-3’) | | Product size | Amplification reaction conditions |
| --- | --- | --- | --- | --- | --- |
| *MUT* | E2 | E2F | tcctatttcccaccccctct | 700 | 95℃ 5 min, 94℃ 30 s, 62℃ 45 s, 72℃ 1 min, 20cycls; 95℃ 5 min, 94℃ 30 s, 58℃ 45 s, 72℃ 1 min, 15cycls |
|  |  | E2R | atcatctttacagagattaaccccc |  |  |
|  | E3 | E3F | tggaataactgctccattcatagtc | 398 |  |
|  |  | E3R | ggcacaagggaaacaactgac |  |  |
|  | E5 | E5F | ccagatgtctgacagtgtgct | 249 |  |
|  |  | E5R | agaccttgattttctagtgtgtga |  |  |
|  | E6 | E6F | agaacctccccaaggatcag | 293 |  |
|  |  | E6R | tgcagcagttgctgtttaatc |  |  |
|  | E12 | E12F | ttctcccattctgtaggt | 461 |  |
|  |  | E12R | actcaagattcccatcac |  |  |
| *MMACHC* | E1 | E1F | ggaaatggagtcgaagctga | 246 |  |
|  |  | E1R | ttgcaacgaagccaatcata |  |  |
|  | E3 | E3F | tcccctcatgctgacagtac | 209 |  |
|  |  | E3R | tttccctctcacctggttcc |  |  |
|  | E4 | E4F | ccagaaaacctcatgactgtgta | 213 |  |
|  |  | E4R | agggagaactaggcttctctga |  |  |
| *MCEE* | E2 | E2F | tccatttatctcctggtttc | 842 |  |
|  |  | E2R | tgtttctcctaattgcctta |  |  |
